# Analysis of Mycotoxins and Cytotoxicity of Airborne Molds Isolated from the Zoological Garden—Screening Research

**DOI:** 10.3390/pathogens13040294

**Published:** 2024-03-30

**Authors:** Kinga Plewa-Tutaj, Magdalena Twarużek, Robert Kosicki, Ewelina Soszczyńska

**Affiliations:** 1 Department of Microbial Ecology and Acaroentomology, Faculty of Biological Sciences, University of Wrocław, 51-148 Wrocław, Poland; 2 Department of Physiology and Toxicology, Faculty of Biological Sciences, Kazimierz Wielki University, 85-064 Bydgoszcz, Poland; twarmag@ukw.edu.pl (M.T.); robkos@ukw.edu.pl (R.K.); eweso@ukw.edu.pl (E.S.)

**Keywords:** MTT assay, mycotoxins, HPLC

## Abstract

Objective: The objective of this paper was to assess the airborne mold contamination, secondary metabolite profiles, and cytotoxicity of the dominant fungal species isolated from the air in selected rooms at a Zoological Garden. Materials and methods: Fungal concentrations were measured with MAS-100 air samplers. The collected airborne fungi were identified using a combination of morphological and molecular methods. The cytotoxicity of 84 strains belonging to two *Penicillium* and *Aspergillus* genera was determined using the quantitative colorimetric MTT (3-(4,5-dimethylthiazol-2-yl)-2,5-diphenyltetrazolium salt) assay. The mycotoxins were detected using high-performance liquid chromatography (HPLC) with a mass spectrometry detector. Results: The ITS gene was amplified and sequenced to identify the 132 species. For mycotoxicological and cytotoxicity analyses, 52 *Penicillium* isolates and 32 *Aspergillus* representatives were selected. Cytotoxicity was confirmed in 97.6% of cases analyzed. Using the LC-MS/MS method, 42 out of 84 strains produced at least one of the following toxins: ochratoxin A, ochratoxin B, patulin, gliotoxin, roquefortine C, griseofulvin, sterigmatocystin, fumonisin B2, moniliformin, and mycophenolic acid. Conclusions: Analytical methods for assessing the presence of mycotoxins in fungal isolates collected directly from the air have proven to be an effective tool. Our research provides new information on the occurrence of potentially toxin-producing molds within a zoo.

## 1. Introduction

Mycotoxins are secondary metabolites produced by filamentous fungi, particularly molds belonging to the *Aspergillus*, *Fusarium*, *Penicillium*, *Claviceps*, and *Alternaria* genera. The term refers to a group of small and stable molecules, including alkaloids, cyclopeptides, coumarins, aromatic and phenolic structures, and terpenoids [1]. Currently, there are over 100,000 known fungal species, with more than 500 characterized mycotoxins [2]. Of these, around 30 have been identified as harmful to human and animal health [3]. These mycotoxins include aflatoxins, citrinin, ochratoxins, patulin, trichothecenes (i.a., nivalenol, deoxynivalenol, T-2 toxin, HT-2 toxin), zearalenone, fumonisins, and ergot alkaloids like ergotamine [4]. In the general population, exposure to mycotoxins occurs primarily through the food chain by ingestion of contaminated food. In addition to the dietary route, some people may be exposed to mycotoxins by inhalation, mainly in the workplace or in water-damaged buildings, through the growth of mycotoxigenic molds on materials and products. However, the possible presence of mycotoxins in the air is less well documented, especially in the air of zoos [1].

Most mycotoxins are not volatile. Nevertheless, they may be present in airborne dust and fungal spores, as well as other smaller fragments of these microorganisms. Interestingly, a species can produce more than one toxin, and a particular toxin is not restricted to a single genus, species, or strain. Furthermore, the production of mycotoxins by a fungal colony varies with environmental conditions. Mycotoxins may be present in the environment even in the absence of visible fungi. On the other hand, the detection of toxigenic fungal species does not indicate that they are capable of producing these metabolites [1]. Therefore, research on the assessment of mycological air quality in public spaces, such as the zoological garden studied, should not be limited to the detection of airborne fungi but should also include, for example, mycotoxins determination and cytotoxicity studies. These methods would allow the possibility of mycotoxin production by potentially toxic species to be assessed, as well as their toxic effect on cells. Such an extended quantitative and qualitative analysis of airborne molds will provide a preliminary picture of mycological hazards. This is important, for example, to determine occupational and potential risks to employees. Therefore, our aim was to determine the airborne mold contamination, secondary metabolite profiles, and cytotoxicity of the dominant fungal species isolated from the air of the selected rooms in the Zoological Garden.

## 2. Materials and Methods

### 2.1. Study Area and Sampling Method 

The study was conducted at the Zoological Garden in Wroclaw in 20 facilities: Monkey House (two sites), Apes Pavilion (four sites), Papio Pavilion (one site), Maggots Pavilion (three sites), Kongo Pavilion (five sites), East Africa Pavilion (five sites). The selection of the study site was based on its convenient accessibility.

Air samples were taken in every season (spring, summer, autumn, winter) using a MAS-100 air sampler (Merck KgaA, Darmstadt, Germany). At the central point of each place, three parallel samples (two incubated in 27 °C degree, one in 37 °C) were taken and impacted onto the Sabouraud agar surface. The samples were incubated at 25 °C for 7 days (two parallel) and at 37 °C for 5 days (one parallel). The probe’s air flow rate is 11 m/s, and the sampling frequency is 100 L/min. In our research, we consistently collected 20 L of air, which is the experimentally determined volume and the largest possible volume for mycological analysis of the studied object. After incubation, the number of colonies of fungi was expressed as a colony-forming unit (CFU) per 1 m^3^ of the air (CFU/m^3^). 

### 2.2. Morphological and Molecular Identification of Molds

All isolated molds were identified using diagnostic keys [5,6,7,8] based on macroscopic and microscopic observations of the fungal colonies. After that, isolated strains that were difficult to diagnose underwent molecular identification. DNA extraction was performed using the Tissue DNA Purification Kit (EURx) according to the manufacturer’s instructions. Molecular analyses are based mainly on the sequence of the internal transcribed spacer ITS. The ITS was amplified using a pair of primers: ITS1 (5′-TCCGTAGGTGAACCTGCGG-3′) and ITS4 (5′-TCCTCCGCTTATTGATATGC-3′). PCR reactions were performed in a T100 Thermal Cycler (Bio-Rad, Warsaw, Poland) in a total volume of 12.5 µL containing 6.25 μL of 2 × PCR Mix Plus (A@A Biotechnology, Gdansk, Poland), 0.625 μL of each primer (10 mM), 4 μL of DNA template and 1 μL of ddH2O. PCR conditions included an initial denaturation step of 95 °C (30 s), 34 cycles of 95 °C (45 s), 55 °C (60 s) and 72 °C (60 s), and the final elongation of 72 °C (3 min). PCR products were separated by electrophoresis on a 1% agarose gel stained with SimplySafe (EURx, Gdansk, Poland). All the PCR-positive samples were purified and sequenced (Macrogen, Amsterdam, Netherlands) with the primers used for DNA amplification. The nucleotide sequences obtained in this study were manually edited by the use of the DNA Baser Sequence Assembly software (Heracle BioSoft SRL, Romania), software and consensus sequences were aligned and compared with those deposited in the GenBank of the National Center for Biotechnology Information (NCBI, Bethesda, MD, USA) using the BLAST algorithm. The isolates’ sequences have been deposited in the GenBank database (Supplemental Table S1).

### 2.3. MTT Test for Assessing Fungal Cytotoxicity

The cytotoxicity studies were performed using the quantitative colorimetric MTT (3-(4,5-dimethylthiazol-2-yl)-2,5-diphenyltetrazolium salt) test. This test can be used to evaluate the cytotoxicity of various types of materials, for example, molds and their metabolites, bacterial cultures, feed, or food. The principle of the test is based on the conversion of yellow tetrazolium salt (MTT) into purple water-insoluble formazan. This conversion takes place in living cells [9]. The cytotoxicity studies were performed using the quantitative colorimetric MTT (3-(4,5-dimethylthiazol-2-yl)-2,5-diphenyltetrazolium salt) test. This test can be used to evaluate the cytotoxicity of various types of materials, for example, molds and their metabolites, bacterial cultures, feed, or food. The principle of the test is based on the conversion of yellow tetrazolium salt (MTT) into purple water-insoluble formazan. This conversion takes place in living cells [9].

The cells were cultured in MEM (Minimum Essential Medium Eagle; Sigma-Aldrich, St. Louis, MI, USA) supplemented with an antibiotic solution (stock solution: 10,000 units of penicillin and 10 mg of streptomycin per mL in 0.9% NaCl (Sigma-Aldrich, St. Louis, MI, USA), and 5% fetal calf serum (Sigma-Aldrich, St. Louis, MI, USA) in a CO^2^-incubator (CB, BINDER GmbH, Tuttlingen, Germany) (5% CO^2^, 37 °C, 98% humidity). The use of these cells is an alternative to primary human cells for renal in vitro toxicology due to their high similarity in renal physiology [10]. In addition, these cells are characterized by high sensitivity to the most common mycotoxins.

For the initial stage of the cytotoxicity assessment procedure, the fungi were incubated on Chapek-Dox agar medium (Sigma-Aldrich, St. Louis, MI, USA) at 28 °C for two weeks. Then, they were transferred to a temperature of 4 °C for the following 10 days. The fungi were inoculated to cover the entire surface of the medium on 90 mm Petri dishes. For mold extraction, the content of a whole plate was transferred to a mixing bag (BagLight^®^ 400, Intersciences, France). Then, 50 mL chloroform was added, and bags were treated for 5 min in a bag mixer (BagMixer^®^ 400, Intersciences, France). Chloroform was used to prepare the extracts. Subsequently, sample extracts were filtered through a paper filter (Whatman™, 59512, 185 mm in diameter) into a round bottom flask and evaporated to dryness in a vacuum evaporator. The evaporation residue was dissolved in 2 mL of chloroform using an ultrasonic bath and pipetted into the vials, then the extracts were evaporated to dryness under a gentle flow of nitrogen at 30 C. Prior to the MTT test, extracts were dissolved in 1 mL of mixture of ethanol dimethyl sulfoxide-minimum essential medium with Earle’s salts (MEM) (1.7 + 0.3 + 98, *v*/*v*/*v*) as described by Hanelt et al. 1994 [11]. Then, serial log 2 dilutions of sample extract were prepared (1–31.5 cm^2^/mL, 2–15.625 cm^2^/mL, 3–7.813 cm^2^/mL, 4–3.906 cm^2^/mL, 5–1.953 cm^2^/mL, 6–0.977 cm^2^/mL, 7–0.488 cm^2^/mL, 8–0.244 cm^2^/mL, 9–0.122 cm^2^/mL, and 10–0.061 cm^2^/mL). All plates were incubated at 37 °C in a humidified atmosphere with 5% CO^2^ for 48 h. Then, MTT stock solution (20 µL) was added to each well, and plates were incubated for another 4 h. After removing the supernatant with a multichannel micropipette, 100 µL DMSO was added to each well, and absorbance was measured spectrophotometrically with an ELISA-Reader microplate reader (ELISA LEDETECT 96, Biomed Dr. Wieser GmbH, Salzburg, Austria) at a wavelength of 510 nm (=maximum absorption wavelength of formazan derivatives).

If the absorbance was less than 50% of the cell division activity, all samples analyzed were considered toxic. A semi-quantitative scale was used to grade cytotoxicity. A low cytotoxic effect (+) was observed for IC_50_ values ranging from 31.251 cm^2^/mL to 7.813 cm^2^/mL, a medium cytotoxic effect (++) for values ranging from 3.906 cm^2^/mL to 0.977 cm^2^/mL, and a high cytotoxic effect (+++) for values ranging from 0.488 cm^2^/mL to 0.061 cm^2^/mL. The absence of cytotoxicity was determined when the absorbance value was ≥50 for the first dilution tested (31.5 cm^2^/mL). The cytotoxicity studies were performed at the Faculty of Biological Sciences, Kazimierz Wielki University, Bydgoszcz, Poland.

### 2.4. High-Performance Liquid Chromatography (HPLC)

The procedure for sample preparation and chromatographic analysis of mycotoxins was followed according to Pietrzak et al. (2015) [12]. Before conducting chromatographic analyses, the mold isolates were incubated at 28 °C for approximately two weeks on Czapek-Dox agar medium (Sigma-Aldrich). Subsequently, the samples were transferred to a temperature of 4 °C for the following 10 days. Due to the diffusion of mycotoxins into the medium and the difficulty of collecting all the mycelia from the Petri dish, analysis of pure fungal material alone would not accurately reflect the total amount of mycotoxins formed. Therefore, we decided to homogenize the entire fungus with the medium and take an equal mass for each sample. Briefly, 2.0 g of sample (homogenized entire content of the plate) was vigorously shaken for 60 min with 8.0 mL of an acetonitrile/water/acetic acid mixture (79/20/1; *v*/*v*/*v*). After centrifugation for 10 min at 7000 rpm, 40 µL of the extract was diluted with 960 µL of a methanol/water mixture (2/8; *v*/*v*) and centrifuged again for 30 min at 14,500 rpm. The sample dilution factor was 100.

Mycotoxin detection was carried out using a high-performance liquid chromatography (HPLC) system, specifically the Nexera model from Shimadzu (Kyoto, Japan), coupled with a mass spectrometry detector, the 5500 QTrap from Sciex (Foster City, USA). The mycotoxins were separated chromatographically on a Gemini C18 column (150 × 4.6 mm, 5 μm) manufactured by Phenomenex (Torrance, CA, USA) at a flow rate of 1 mL/min, with an injection volume of 5 μL.

Two mobile phases were used in this experiment. Phase A was composed of methanol, water, and acetic acid in a ratio of 10:89:1 (*v*/*v*/*v*), while Phase B was composed of methanol, water, and acetic acid in a ratio of 97:2:1 (*v*/*v*/*v*). Both mobile phases were supplemented with 5 mmol/L of ammonium acetate. The chromatographic gradient was as follows: initial elution with 0% B up to 2.0 min, followed by a linear increase to 50% B from 2.0 to 5.0 min, a further increase to 100% B from 5.0 to 14.0 min, maintaining 100% B until 18.0 min, and finally returning to the initial composition of 0% B at 22.5 min.

Tandem mass spectrometry analysis was performed in scheduled multiple reaction monitoring (sMRM) mode for both negative and positive polarities within a single chromatographic run. The electrospray ionization (ESI) source parameters were set as follows: a curtain gas at 30 psi, collision gas at a medium level, ion spray voltage at −4500 V (negative polarity) and 5500 V (positive polarity), ion source temperature maintained at 550 °C, ion source gas1 at 80 psi and ion source gas2 at 80 psi. Table 1 shows the instrument settings optimized for the product ions of each compound. Data acquisition and processing were performed using Analyst 1.6.2 software (Sciex, Foster City, CA, USA).

The concentrations of mycotoxins were determined through external calibration. To determine the limits of detection (LOD) and limits of quantitation (LOQ) for individual mycotoxins, a blank sample extract was spiked with mycotoxin standards. The LOD and LOQ values were calculated using signal-to-noise (S/N) ratios of 3:1 and 10:1, respectively, with the aid of mass spectrometry software (Table 2).

A total of 37 different mycotoxins and secondary metabolites (nivalenol, deoxynivalenol, monoacetoxyscirpenol, diacetoxyscirpenol, T-2 toxin, HT-2 toxin, aflatoxins (M1, B1, B2, G1, G2), patulin, fusarenon X, T-2 tetraol, α-zearalanol, β-zearalanol, α-zearalenol, β-zearalenol, 15-acetyldeoxynivalenol, 3-acetyldeoxynivalenol, deepoxy-deoxynivalenol, fumonisin (B1, B2, B3), zearalenone, zearalenone, T-2 triol, griseofulvin, moniliformin, mycophenolic acid, neosolaniol, ochratoxins (A, B), roquefortine C, sterigmatocystin, gliotoxin, mevinolin) were determined using this method.

## 3. Results and Discussion

Zoos are a popular tourist and educational attraction, with up to one million visitors per year. However, staff are exposed to mycotoxins and bioaerosols produced by the animals. Despite this, there have been few studies on microbial contamination in zoos. Furthermore, none of the studies have addressed the qualitative composition of the molds or their potential toxicity.

Different concentrations of airborne fungi were noted in each season for each location. The highest colony concentration was reported in autumn (3.65 × 10^5^ CFU/m^3^), followed by 1.5 × 10^4^ and 1.32 × 10^4^ CFU/m3 found in spring and summer, respectively. The lowest concentration of fungi (2.5 × 10^2^ CFU/m^3^) was noted in the winter season. The concentration of airborne fungi can increase suddenly due to optimal temperature and high relative humidity. Therefore, during autumn, when meteorological conditions were most favorable for the development and spread of microorganisms, fungi were more abundant than in winter, when humidity and temperature were lower.

Several studies have investigated microbial and mycological air pollution in animal production facilities [13] and agricultural environments [14,15]. However, research on non-production facilities, such as zoological gardens, is scarce. In Poland, Grzyb and Lenart-Boroń (2019) conducted the first study in a zoological garden, which revealed total fungal concentrations similar to those found in our survey (ranging from 8.4 × 10^2^ to 2.84 × 10^4^) [16]. However, the mycological quality of air in zoos has not been extensively researched. Therefore, we compared our findings with those of other breeding facilities, such as poultry houses and cowsheds. Matković et al. (2007) reported that the concentration of fungi in the barn ranged from 5.23 × 10^4^ CFU/m^3^ (at midday) to 8.35 × 10^4^ CFU/m^3^ (in the morning) [17]. Radon et al. (2002) described the air quality in agricultural buildings in Switzerland, Denmark, Germany, and Spain and reported that the total number of fungi in poultry houses and pig farms exceeded the concentrations of airborne fungi found in the study, ranging from 8.3 × 10^4^ to 1.1 × 10^9^ [18].

A high percentage of *Penicillium* and *Aspergillus* genera were observed in all facilities, including Monkey House, Apes Pavilion, Papio Pavilion, Maggots Pavilion, Kongo Pavilion, and East Africa Pavilion. *Aspergillus* and *Penicillium* represented between 24.3% and 61.36% of all isolated fungal genera. Other genera made up a small percentage (between 0.89% and 3.57%) of the molds that were isolated. Therefore, representatives of these two genera were selected for further research.

For cytotoxicity and mycotoxicological analyses, 52 isolates belonging to the *Penicillium* genera and 32 representatives of the *Aspergillus* genera were selected from the 132 strains isolated in our own research. Table 3 and Table 4 show the results of the MTT cytotoxicity test for 83 mold strains. Cytotoxicity was confirmed for the strains belonging to the *Penicillium* and *Aspergillus* genera. The analyzed fungi showed cytotoxicity in 82 cases (97.6%), with only two isolates showing no cytotoxicity. 98.07% of the *Penicillium* strains studied exhibited cytotoxicity, with 29 (55.7%) demonstrating medium to high levels of cytotoxicity. Among the 32 tested *Aspergillus* genera, 30 were found to be cytotoxic, with 23 strains (71.88%) exhibiting medium to high levels of cytotoxicity. The strains with the highest cytotoxicity levels were *A. westerdijikiae* (11al, 1dz), *A. ostianus* (17cz), *A. giganteus* (19cz), *A. fumigatus* (5bw, 4gw, 3fw, 9dl, 10bl), *P. griseofulvum* (17az, 18cz, 19dz), *P. chrysogenum* (15aw), *P. citrinum* (15dl), *P. steckii* (18cw) and *P. sumatraense* (15bz). 

Only a small number of studies have investigated the health outcomes resulting from exposure to airborne mycotoxins. Most of these studies have focused on the cytotoxic effects of samples on cell lines using the MTT assay [19,20,21]. However, due to the lack of research on the cytotoxicity of molds isolated from zoos and breeding facilities, it is not possible to conduct a comparative analysis. Similar studies are mainly concerned with the cytotoxicity of fungi belonging to *Penicillium*, *Stachybotrys*, *Chaetomium*, and *Aspergillus* genera, which are commonly found in various domestic environments [9]. Other research has also been conducted in various hospital wards, with a focus on the group of fungi belonging to the genus *Aspergillus* [22], as well as in museums, composting plants, and tanneries [18,22]. Skóra et al. (2017) identified high percentages of the genera *Alternaria*, *Aspergillus*, and *Penicillium* [19]. The most cytotoxic species were *Alternaria alternata*, *A. limoniasperae*, *Aspergillus flavus*, *Penicillium biourgeianum*, *P. commune*, and *P. spinulosum*. As previously stated, there is a lack of comprehensive microbiological and mycotoxicological studies conducted in zoos. Therefore, our preliminary study provides initial information on the species composition of molds found in these facilities. This study is important because it highlights the potential for airborne fungi to produce mycotoxins.

Molds identified in our study may be associated with allergic respiratory diseases. The genus *Penicillium*, which was most abundant in the zoo air environment, is on the list of the most common allergenic taxa and has been linked to asthma. In addition, the identified *A. flavus* is considered an opportunistic animal and human pathogen, while *A. fumigatus* is the main cause of invasive pulmonary aspergillosis. Some of the identified species are important producers of mycotoxins, as demonstrated in our study. Using the LC-MS/MS method, 42 of the 83 strains produced at least one of the following toxins (ng g-1): ochratoxin A (23.6–257), ochratoxin B (20.3–65.2), patulin (59–491,000), gliotoxin (24.1–4190), roquefortine C (6.06–14,020), griseofulvin (8220–13,600), sterigmatocystin (6.658–29,050), fumonisin B2 (30.93–127.9) moniliformin (8500–52,900), mycophenolic acid (242–63,500). Of the 52 *Penicillium* isolates tested for their ability to produce different types of mycotoxins, 15 (28.8%) strains were positive (Table 5 and Table 6). Three isolates of *Penicillium* griseofulvum produced patulin. This is the most common mycotoxin found in apples, juice, cider, many vegetables, cereals, moldy fruit, and other foods [23]. Patulin has also been detected in some strains isolated from air samples [24]. Patulin has attracted global attention because of the health risks it exacerbates. Indeed, this mycotoxin has mutagenic, neurotoxic, immunotoxic, genotoxic, and gastrointestinal effects in humans and animals [25]. Another secondary metabolite detected in our research was roquefortine C. This toxin was produced by 12 *Penicillium* species, including *P. commune* (3 strains), *P. solitum*, *P. chrysogenum* (2 strains), *P. lanosocoeruleum* (2 strains), and *P. allii* and *Penicillium* sp. (3 strains). Roquefortine C has bacteriostatic activity against Gram-positive microorganisms, neurotoxic properties in chicks and mice, and low acute cytotoxicity on human intestinal cells compared to other mycotoxins. On the other hand, roquefortine C has also been reported to be highly toxic by inhalation [26]. Only one isolate of *P. griseofulvum* produced moniliformin. Moniliformin is a mycotoxin produced by a number of *Fusarium* species. The list of MON-producing species includes *F. acuminatum*, *F. avenaceum*, *F. chlamydosporum*, *F.culmorum*, *F. dlamini*, *F. equiseti*, *F. fusaroides*, *F. semitectum*, *F. sporotrichoides*, *F. fujikuroi*, *F. oxysporum*, *F. proliferatum*, *F. sambucinum*, *F. temperatum*, *F. thapsinum*, *F. subglutians* and *F. tricinctum* [27,28]. Furthermore, MON has been observed to be produced by *Penicillium melanoconidium*. This fungus is capable of producing significant amounts of this mycotoxin [29]. In our study, we show for the first time that moniliformin can also be produced by airborne *P. griseofulvum*. However, this result will be re-evaluated in further studies to determine the precise taxonomic position and metabolic profile of the fungus. This species is mainly known for its production of griseofulvin and patulin, which was demonstrated in our work. Eleven isolates belonging to the *Penicillium* genera (*P. chrysogenum*, *P. glabrum*, *P. lanosocoeruleum*, *P. commune*, *P. glandicola* and *Penicillium* sp.) produced mycophenolic acid and several isolates (*P. griseofulvum*, *P. raistrickii*, *P. chrysogenum*, *P. lanosocoeruleum*) produced high concentrations of griseofulvin. Unlike the mycotoxins already described, the last fungal metabolite can have a beneficial effect on human health. For example, griseofulvin is used to treat dermatophytosis in both humans and animals. Recently, this mycotoxin has attracted attention due to reports of its potential anticancer effects [30]. Mycophenolic acid (MPA), on the other hand, is an antibiotic and immunosuppressant. MPA and its derivatives also have diverse biological properties, such as antineoplastic, anti-inflammatory, anti-psoriasis, antifungal, and antiviral activities. Recent reports indicate anti-SARS-CoV-2 activity of MPA [31].

Out of the 32 *Aspergillus* isolates that were tested for their ability to produce mycotoxins, 23 (71.88%) strains were positive (Table 7 and Table 8). Among the nine *A. fumigatus* strains that were examined, eight produced gliotoxin, and three strains produced roquefortine C. While roquefortine C is considered toxic to humans and animals and has been associated with several health risks, including neurotoxicity [32], gliotoxin (GT) has been implicated as a virulence factor in human and animal aspergillosis. Its functions are to inhibit the phagocytic activity of macrophages, reduce the cytotoxic activity of T-cells, and prevent the induction of apoptosis in host cells. Additionally, studies have shown a positive correlation between GT production and the pathogenicity of *A. fumigatus* [33]. None of the *Aspergillus* isolates produced aflatoxins. However, two isolates from the *Aspergillus* genus (*A. sydowii, A. puulaensis*) and one from the *Penicillium* genus (*P. commune*) were capable of producing sterigmatocystin. Inhalation of air contaminated with sterigmatocystin can cause respiratory symptoms, and long-term exposure has been linked to liver and lung cancer. This mycotoxin is classified by the IARC (International Agency for Research on Cancer) as possibly carcinogenic to humans (Group 2B) (IARC Working Group on the Evaluation of Carcinogenic Risks to Humans [34]. In vivo studies have shown that sterigmatocystin is capable of inducing toxic effects in several animals, including fish, ruminants, chickens, mice, rats, pigs, and monkeys [35]. Ochratoxin A and B were produced by one strain of *A. niger* and two strains of *A. elegans*. Ochratoxin A (OTA) is a toxic metabolite with nephrotoxic and carcinogenic effects. In addition, various studies have shown that OTA is also hepatotoxic, embryotoxic, teratogenic, neurotoxic, immunotoxic, and genotoxic in many species. Furthermore, OTA has been classified by the International Agency for Research on Cancer (IARC) as a possible human carcinogen (Group 2B) [36]. Ochratoxin B (OTB) is the non-chlorinated analog of the mycotoxin ochratoxin A (OTA). Although closely related in structure, OTB is considered to be much less toxic [3]. Three *A. niger* strains demonstrated the ability to produce fumonisin B. Fumonisins are secondary metabolites produced mainly by *Fusarium verticillioides* and *Fusarium proliferatum* [37]. Recently *Aspergillus niger* has been reported to be a fumonisin B2 (FB2) producer [38]. Fumonisin B toxins, as structural analogs of sphingoid bases, inhibit ceramide synthases, causing the disruption of the sphingolipid metabolism and leading to sphinganine (and sphingosine) accumulation in cells and tissues. Toxicity studies have mainly focused on the effects of FB1, but FB2 appears to have similar toxicological profiles. 

Most animal housing facilities provide good humidity and temperature conditions for fungal growth. They are, therefore, considered critical for worker exposure to airborne fungi. The main sources of information on occupational exposures to airborne mycotoxins are studies in settings related to livestock and animal production [39,40,41], farming [19,42], and waste and sewage treatment plants [43,44,45]. However, studies of mycotoxin exposure in this area have been relatively scarce. In addition, no similar studies have been conducted in zoos. Concentrations of cultivable bacteria and fungi, and in some cases endotoxins, are usually monitored to assess biological risks in the workplace. However, some researchers have started the measurement of biological agents with potential effects on human health, such as mycotoxins, in the workplace. But this has never been performed routinely. Several studies demonstrated the existence of occupational AFB1 exposure in poultry and pig farms in Portugal [46,47]. In dust samples collected from poultry farms, Skóra et al. (2016) detected the mycotoxins typical of *Fusarium* spp: 15-hydroxyculmorin, apicidin, aurofusarin, α-zearalenol, β-zearalenol, deoxynivalenol, epi-equisetin, equisetin, zearalenone and zearalenone sulfate [40]. A potential health concern for OTA and FB was also identified by biomonitoring in animal production farms (poultry, pig, dairy) [36]. Other researchers have noted that feed handling is a crucial task in pig production plants. In these working environments, high levels of contamination have been found in litter (DON: <LOQ–76.4 ng/g; STC: 1.14–2.69 ng/g) and feed samples. It is estimated that feeding, floor sweeping, and removal/change of litter are responsible for the workers’ exposure to dust and mycotoxins [37].

Mycotoxins are well-known for their cytotoxic effects and have been extensively studied due to their potential health hazards. These toxic secondary metabolites are produced by certain fungi as a defense mechanism, and when ingested or inhaled by humans or animals, they can disrupt cellular processes and cause cellular damage or death. Common examples of mycotoxins include aflatoxins, ochratoxin A, patulin, and fumonisins, each with its own specific cytotoxic mechanisms and target organs [48].

Fungi produce a diverse array of secondary metabolites beyond mycotoxins, many of which have been shown to possess bioactive properties. These metabolites include enzymes, organic acids, volatile organic compounds, and various other compounds with potential biological activities. While not as extensively studied as mycotoxins, some of these metabolites have been implicated in cytotoxicity and other health effects. For instance, certain organic acids produced by fungi may disrupt cellular membranes or metabolic pathways, leading to cytotoxic effects [49].

In our study, cytotoxicity was observed in most of the fungi tested. However, it should be noted that only a small number of them produced limited amounts of mycotoxins. The interaction between different mycotoxins and other fungal metabolites can lead to synergistic or cumulative effects on cytotoxicity. When multiple toxic compounds are present simultaneously, they may act together to exacerbate cellular damage beyond what would be expected from each compound individually [50,51]. This synergism can occur through various mechanisms, such as enhancing cellular uptake or interfering with detoxification pathways. Additionally, cumulative exposure to multiple toxins over time can lead to chronic health effects, even at relatively low concentrations.

The overall cytotoxic effects observed in fungal cultures are likely due to a combination of mycotoxins, other fungal metabolites, and possibly interactions with host factors. This complexity underscores the challenges in assessing and mitigating the health risks associated with fungal contamination. It also emphasizes the need for comprehensive approaches that consider the full spectrum of fungal metabolites and their potential interactions. 

In conclusion, while mycotoxins are significant contributors to cytotoxicity, other fungal metabolites also play a role in mediating cellular damage. Understanding the interplay between different toxic compounds produced by fungi is essential for accurately assessing the health risks associated with fungal contamination and developing effective strategies for prevention and mitigation.

## 4. Conclusions

The impact of airborne mycotoxins on human health is not yet fully understood, although there is increasing interest in their role in the occupational environment. Our research presents new information on the presence of molds that may produce toxins in a particular environment, specifically a zoological garden. However, to fully understand the airborne fungal composition in the zoological garden and its potential negative impact on human and animal health, further long-term quantitative, qualitative, and mycotoxicological research is necessary in all animal enclosures.

The analytical methods employed, namely cytotoxicity assessment and chromatographic analysis, have proven to be effective tools for assessing the presence of mycotoxins in fungal isolates collected directly from the air. The studies indicate that workers may be exposed to dangerous and potentially cancer-causing mycotoxins, such as sterigmatocystin and ochratoxin. However, it is important to note that there are limitations to these studies. Regarding health outcomes, these studies are insufficient to provide a clear picture of the health risks associated with inhalation of mycotoxins. Therefore, further field research is necessary to identify the work tasks that pose the highest risk of exposure to mycotoxins through inhalation. This information will be crucial for health professionals responsible for implementing prevention and control strategies. In addition, researchers should collaborate on developing standardized sampling and analysis methods, as well as conducting large-scale studies.

## Figures and Tables

**Table 1:** Optimized electrospray ionization tandem mass spectrometry (ESI)-MS/MS conditions for analytical method.

		Precursor Ion [*m*/*z*]	Product Ions [*m*/*z*] ^a^	Declustering Potential [V]	Collision Energy [V]	Cell exit Potential [V]
15-Acetyldeoxynivalenol	[M+H]+	339.1	321.2/137.2	91	13/17	18/8
3-Acetyldeoxynivalenol	[M+Ac]−	397.3	59.2/307.1	−70	−38/−20	−8/−7
Aflatoxin B1	[M+H]+	313.1	285.2/128.1	106	33/91	16/10
Aflatoxin B2	[M+H]+	315.1	287.2/259.2	96	37/43	18/18
Aflatoxin G1	[M+H]+	329.1	243.1/200.0	86	39/59	14/12
Aflatoxin G2	[M+H]+	331.1	313.2/245.2	111	35/43	18/14
Aflatoxin M1	[M+H]+	329.1	273.2/229.1	91	35/59	16/12
a-Zearalanol	[M-H]−	321.2	277.2/303.2	−115	−32/−30	−13/−15
a-Zearalenol	[M-H]−	319.2	160.1/130.1	−115	−44/−50	−13/−20
b-Zearalanol	[M-H]−	321.2	277.2/303.2	−115	−32/−30	−13/−15
b-Zearalenol	[M-H]−	319.2	160.0/130.0	−115	−44/−50	−13/−20
Deepoxydeoxynivalenol	[M+Ac]−	339.1	59.1/249.0	−70	−20/−18	−9/−17
Deoxynivalenol	[M+Ac]−	355.1	265.2/59.2	−70	−22/−40	−13/−8
Diacetoxyscirpenol	[M+NH4]+	384.2	307.2/105.1	81	17/61	9/7
Deoxynivalenol 3-Glucoside	[M+Ac]−	517.3	427.1/59.1	−80	−30/−85	−11/−7
Fumonisin B1	[M+H]+	722.5	334.4/352.3	121	57/55	4/12
Fumonisin B2	[M+H]+	706.5	336.4/318.4	126	59/51	8/2
Fumonisin B3	[M+H]+	706.5	336.3/318.5	126	59/51	8/2
Fusarenon X	[M+Ac]−	413.2	59.1/263.0	−70	−44/−22	−9/−16
Gliotoxin	[M+H]+	327.1	263.2/245.3	61	15/25	16/20
Griseofulvin	[M+H]+	353.2	165.2/215.2	81	27/27	10/12
HT-2 Toxin	[M+NH4]+	442.2	263.1/345.1	76	21/27	19/20
Mevinolin	[M+H]+	405.3	199.2/173.3	76	17/29	14/10
Moniliformin	[M-H]−	96.9	41,2	−100	−24	−5
Monoacetoxyscirpenol	[M+NH4]+	342.2	265.1/307.2	71	13/13	26/8
Mycophenolic acid	[M+NH4]+	338.1	207.2/303.2	61	33/19	16/18
Neosolaniol	[M+NH4]+	400.2	215.0/185.0	76	25/29	12/14
Nivalenol	[M+Ac]−	371.1	281.1/59.1	−75	−22/−45	−15/−7
Ochratoxin A	[M+H]+	404.0	239.0/102.0	91	37/105	16/14
Ochratoxin B	[M+H]+	370.1	205.0/103.1	86	33/77	12/16
Patulin	[M-H]−	153.0	109.0/81.0	−50	−12/−18	−9/−11
Roquefortine C	[M+H]+	390.2	193.2/322.2	91	39/29	10/18
Sterigmatocystin	[M+H]+	325.1	310.2/281.1	96	35/51	18/16
T-2 Tetraol	[M+NH4]+	316.2	215.2/281.2	61	13/13	16/8
T-2 Toxin	[M+NH4]+	484.3	215.2/185.1	56	29/31	18/11
T-2 Triol	[M+NH4]+	400.2	281.3/215.2	71	13/17	16/12
Zearalenone	[M-H]−	319.2	205.2/107.0	−125	−34/−40	−13/−5
Zearalenone	[M-H]−	317.1	131.1/175.0	−110	−42/−34	−8/−13

^a^ the ions are presented in quantitative and qualitative order.

**Table 2:** Limits of detection (LOD) and quantification (LOQ) for mycotoxins analyzed by LC-MS/MS.

	Limit of Detection LOD (ng/g)	Limit of Quantification (LOQ) (ng/g)
15-acetyldeoxynivalenol	3.6	12.0
3-acetyldeoxynivalenol	4.2	14.0
aflatoxin B1	0.9	3.0
aflatoxin B2	1.5	5.0
aflatoxin G1	1.2	4.0
aflatoxin G2	1.2	4.0
aflatoxin M1	1.5	5.0
deepoxydeoxynivalenol	4.2	14.0
deoxynivalenol	7.8	26.0
deoxynivalenol 3-glucoside	7.5	25.0
diacetoxyscirpenol	1.2	4.0
fumonisin B1	3.6	12.0
fumonisin B2	3.0	10.0
fumonisin B3	3.6	12.0
fusarenon X	6.0	20.0
gliotoxin	2.1	7.0
griseofulvin	1.2	4.0
HT-2 toxin	1.8	6.0
mevinolin	2.4	8.0
moniliformin	15.0	50.0
monoacetoxyscirpenol	1.8	6.0
mycophenolic acid	3.6	12.0
neosolaniol	1.5	5.0
nivalenol	9.0	30.0
ochratoxin A	1.5	5.0
ochratoxin B	1.8	6.0
patulin	6.0	20.0
roquefortine C	1.8	6.0
sterigmatocystin	1.5	5.0
T-2 toxin	0.9	3.0
T-2 tetraol	13.5	45.0
T-2 triol	2.4	8.0
zearalanone	3.3	11.0
zearalenone	1.5	5.0
α-zearalanol	2.1	7.0
α-zearalenol	2.4	8.0
β-zearalanol	3.0	10.0
β-zearalenol	2.1	7.0

**Table 3:** Level of cytotoxicity of mold isolates belonging to *Penicillium* genera obtained from culture on agar.—no cytotoxicity; + low cytotoxicity (31.251 cm^2^/mL to 7.813 cm^2^/mL); ++ moderate cytotoxicity (3.906 cm^2^/mL to 0.977 cm^2^/mL); +++ high cytotoxicity (0.488 cm^2^/mL to 0.061 cm^2^/mL).

Species	Level of Cytotoxicity				IC50	Dilution
					cm^2^/mL	Steps *
*P. commune* 5aw			++		3.906	4
*P. commune* 14bz		+			7.813	3
*P. commune* 22az		+			7.813	3
*P. commune* 17fz		+			31.25	1
*P. commune* 14aj		+			7.813	3
*P. solitum* 15aj		+			31.25	1
*P. raistrickii* 9bw			++		0.977	6
*P. glandicola* 8bw			++		0.977	6
*P. glandicola* 7aw			++		0.977	6
*P. glandicola* 7bw			++		0.488	7
*P. glandicola* 6aw			++		1.953	5
*P. griseofulvum* 17az				+++	0.061	10
*P. griseofulvum* 18cz				+++	0.061	10
*P. griseofulvum* 19dz				+++	0.061	10
*P. chrysogenum* 18bj			++		3.906	4
*P. chrysogenum* 22aj		+			31.25	1
*P. chrysogenum* 20aez		+			15.625	2
*P. chrysogenum* 15aw				+++	0.244	8
*P. glabrum* 1bw			++		0.488	7
*P. glabrum* 4cw		+			7.813	3
*P. glabrum* 9aw			++		1.953	5
*P. citreosulfuratum* 1az		+			31.25	1
*P. lanosocoeruleum* 24aj			++		1.953	5
*P. lanosocoeruleum* 17aj		+			7.813	3
*P. allii* 19ez		+			15.625	2
*P. citrinum* 15az		+			7.813	3
*P. citrinum* 19bz		+			7.813	3
*P. citrinum* 15bl		+			15.625	2
*P. citrinum* 15dl				+++	0.244	8
*P. steckii* 18cw				+++	0.244	8
*P. steckii* 20cz		+			31.25	1
*P. steckii* 21bj			++		3.906	4
*P. steckii* 22ej		+			15.625	2
*P. steckii* 18bz	none				-	-
*P. steckii* 25cj			++		3.906	4
*P. sumatraense* 15bz				+++	0.122	9
*P. sumatraense* 8dl			++		0.977	6
*P. sumatraense* 16dw			++		0.488	7
*P. copticola* 17bz			++		7.813	3
*P. brevicompactum* 30ej			++		1.953	5
*P. brevicompactum* 28 d			++		1.953	5
*P. brevicompactum* 2b		+			7.813	3
*P. bialowieziense* 29a			++		1.953	5
*P. bialowieziense* 9bj		+			31.25	1
*P. olsonii* 25bj			++		3.906	4
*P. olsonii* 13bz		+			15.625	2
*Penicillium* sp. 1cz		+			31.25	1
*Penicillium* sp. 11dl			++		1.953	5
*Penicillium* sp. 12al			++		1.953	5
*Penicillium* sp. 19bj		+			31.25	1
*Penicillium* sp. 19cj		+			31.25	1
*Penicillium* sp. 27bj			++		3.906	4

* Dilution steps (two-fold serial dilution method) until the absorbance values decreased below 50% of the controls.

**Table 4:** Level of cytotoxicity of mold isolates belonging to *Aspergillus* genera obtained from culture on agar.—no cytotoxicity; + low cytotoxicity (31.251 cm^2^/mL to 7.813 cm^2^/mL); ++ moderate cytotoxicity (3.906 cm^2^/mL to 0.977 cm^2^/mL); +++ high cytotoxicity (0.488 cm^2^/mL to 0.061 cm^2^/mL).

Species	Level of Cytotoxicity				IC50	Dilution
					cm^2^/mL	Steps *
*A. ochraceus* 4bw			++		1.953	5
*A. ochraceus* 17ez			++		0.488	7
*A. ochraceus* 2bz		+			31.25	1
*A. ochraceus* 1bl			++		0.488	7
*A. westerdijikiae* 11al				+++	0.244	8
*A. westerdijikiae* 1dz				+++	0.122	9
*A. ostianus* 17cj			++		3.906	4
*A. ostainus* 17cz *A.elegans* 14al		+		+++	15.625	2
*A. elegans* 14cl			++		3.906	4
*A. elegans* 24bz			++		1.953	5
*A. elegans* 22cz			++		3.906	4
*A. flavus* 2gw			++		0.977	2
*A. flavus* 18aw			++		3.906	4
*A. giganteus* 19cz				+++	0.061	10
*A. sydowii* 5bl		+			15.625	2
*A. puulaeansis* 4bl		+			7.813	3
*A. niger* 7fj			++		3.906	4
*A. niger* 15cj	none				-	-
*A. niger* 25aj			++		3.906	4
*A. niger* 2al			++		3.906	4
*A. niger* 21az		+			31.25	1
*A. niger* 25cz		+			31.25	1
*A. fumigatus* 5bw				+++	0.061	10
*A. fumigatus* 4gw				+++	0.122	9
*A. fumigatus* 3fw				+++	0.061	10
*A. fumigatus* 9bl			++		0.977	6
*A. fumigatus* 10al		+			7.813	3
*A. fumigatus* 6cw		+			31.25	1
*A. fumigatus* 9dl				+++	0.0038	14
*A. fumigatus* 10bl				+++	0,0038	14
*A. fumigatus* 6dl			++		3.906	4

* Dilution steps (2-fold serial dilution method) until the absorbance values decreased below 50% of the controls.

**Table 5:** Mycotoxins produced by *Penicillium* sp.

Metabolites	Concentration (ng g^−1^)										
	*P. chrysogenum* 18bj	*P. chrysogenum* 22aj	*P. chrysogenum* 9aw	*P. lanosocoeruleum* 24aj	*P. lanosocoeruleum* 17aj	*P.allii* 19ez	*Penicillium* sp. 11dl	*Penicllium* sp.12al	*Penicillium* sp. 19bj	*Penicillium* sp. 19cj	*Penicillium* sp. 27 bj
15-acetyldeoxynivalenol	<LOD	<LOD	<LOD	<LOD	<LOD	<LOD	<LOD	<LOD	<LOD	<LOD	<LOD
3-acetyldeoxynivalenol	<LOD	<LOD	<LOD	<LOD	<LOD	<LOD	<LOD	<LOD	<LOD	<LOD	<LOD
aflatoxin B1	<LOD	<LOD	<LOD	<LOD	<LOD	<LOD	<LOD	<LOD	<LOD	<LOD	<LOD
aflatoxin B2	<LOD	<LOD	<LOD	<LOD	<LOD	<LOD	<LOD	<LOD	<LOD	<LOD	<LOD
aflatoxin G1	<LOD	<LOD	<LOD	<LOD	<LOD	<LOD	<LOD	<LOD	<LOD	<LOD	<LOD
aflatoxin G2	<LOD	<LOD	<LOD	<LOD	<LOD	<LOD	<LOD	<LOD	<LOD	<LOD	<LOD
aflatoxin M1	<LOD	<LOD	<LOD	<LOD	<LOD	<LOD	<LOD	<LOD	<LOD	<LOD	<LOD
deepoxydeoxynivalenol	<LOD	<LOD	<LOD	<LOD	<LOD	<LOD	<LOD	<LOD	<LOD	<LOD	<LOD
deoxynivalenol	<LOD	<LOD	<LOD	<LOD	<LOD	<LOD	<LOD	<LOD	<LOD	<LOD	<LOD
deoxynivalenol 3-glucoside	<LOD	<LOD	<LOD	<LOD	<LOD	<LOD	<LOD	<LOD	<LOD	<LOD	<LOD
diacetoxyscirpenol	<LOD	<LOD	<LOD	<LOD	<LOD	<LOD	<LOD	<LOD	<LOD	<LOD	<LOD
fumonisin B1	<LOD	<LOD	<LOD	<LOD	<LOD	<LOD	<LOD	<LOD	<LOD	<LOD	<LOD
fumonisin B2	<LOD	<LOD	<LOD	<LOD	<LOD	<LOD	<LOD	<LOD	<LOD	<LOD	<LOD
fumonisin B3	<LOD	<LOD	<LOD	<LOD	<LOD	<LOD	<LOD	<LOD	<LOD	<LOD	<LOD
fusarenon X	<LOD	<LOD	<LOD	<LOD	<LOD	<LOD	<LOD	<LOD	<LOD	<LOD	<LOD
gliotoxin	<LOD	<LOD	<LOD	<LOD	<LOD	<LOD	<LOD	<LOD	<LOD	<LOD	<LOD
griseofulvin	<LOD	<LOQ	<LOD	<LOD	52240	<LOD	550000	430500	<LOQ	<LOQ	<LOD
HT-2 toxin	<LOD	<LOD	<LOD	<LOD	<LOD	<LOD	<LOD	<LOD	<LOD	<LOD	<LOD
mevinolin	<LOD	<LOD	<LOD	<LOD	<LOD	<LOD	<LOD	<LOD	<LOD	<LOD	<LOD
moniliformin	<LOD	<LOD	<LOD	<LOD	<LOD	<LOD	<LOD	<LOD	<LOD	<LOD	<LOD
monoacetoxyscirpenol	<LOD	<LOD	<LOD	<LOD	<LOD	<LOD	<LOD	<LOD	<LOD	<LOD	<LOD
mycophenolic acid	<LOQ	<LOQ	24.2	<LOQ	41.1	<LOD	<LOD	<LOD	<LOQ	<LOQ	209200
neosolaniol	<LOD	<LOD	<LOD	<LOD	<LOD	<LOD	<LOD	<LOD	<LOD	<LOD	<LOD
nivalenol	<LOD	<LOD	<LOD	<LOD	<LOD	<LOD	<LOD	<LOD	<LOD	<LOD	<LOD
ochratoxin A	<LOD	<LOD	<LOD	<LOD	<LOD	<LOD	<LOD	<LOD	<LOD	<LOD	<LOD
ochratoxin B	<LOD	<LOD	<LOD	<LOD	<LOD	<LOD	<LOD	<LOD	<LOD	<LOD	<LOD
patulin	<LOD	<LOD	<LOD	<LOD	<LOD	<LOD	<LOD	<LOD	<LOD	<LOD	<LOD
roquefortine C	2941	2347	<LOD	3.47	53.4	1090	12.5	<LOD	2051	3210	<LOD
sterigmatocystin	<LOD	<LOD	<LOD	<LOD	<LOD	<LOD	<LOD	<LOD	<LOD	<LOD	<LOD
T-2 toxin	<LOD	<LOD	<LOD	<LOD	<LOD	<LOD	<LOD	<LOD	<LOD	<LOD	<LOD
T-2 tetraol	<LOD	<LOD	<LOD	<LOD	<LOD	<LOD	<LOD	<LOD	<LOD	<LOD	<LOD
T-2 triol	<LOD	<LOD	<LOD	<LOD	<LOD	<LOD	<LOD	<LOD	<LOD	<LOD	<LOD
zearalanone	<LOD	<LOD	<LOD	<LOD	<LOD	<LOD	<LOD	<LOD	<LOD	<LOD	<LOD
zearalenone	<LOD	<LOD	<LOD	<LOD	<LOD	<LOD	<LOD	<LOD	<LOD	<LOD	<LOD
α-zearalanol	<LOD	<LOD	<LOD	<LOD	<LOD	<LOD	<LOD	<LOD	<LOD	<LOD	<LOD
α-zearalenol	<LOD	<LOD	<LOD	<LOD	<LOD	<LOD	<LOD	<LOD	<LOD	<LOD	<LOD
β-zearalanol	<LOD	<LOD	<LOD	<LOD	<LOD	<LOD	<LOD	<LOD	<LOD	<LOD	<LOD
β-zearalenol	<LOD	<LOD	<LOD	<LOD	<LOD	<LOD	<LOD	<LOD	<LOD	<LOD	<LOD

<LOD, below limit of detection; <LOQ, below limit of quantification; values are provided in Table 2.

**Table 6:** Mycotoxins produced by *Penicillium* sp.

Metabolites	Concentration (ng g^−1^)								
	*P. commune* 14bz	*P. commune* 17fz	*P. commune* 14aj	*P. solitum* 15aj	*P. raistrickii* 9bw	*P. glandicola* 7aw	*P. griseofulvum* 17az	*P. griseofulvum* 18cz	*P. griseofulvum* 19dz
15-acetyldeoxynivalenol	<LOD	<LOD	<LOD	<LOD	<LOD	<LOD	<LOD	<LOD	<LOD
3-acetyldeoxynivalenol	<LOD	<LOD	<LOD	<LOD	<LOD	<LOD	<LOD	<LOD	<LOD
aflatoxin B1	<LOD	<LOD	<LOD	<LOD	<LOD	<LOD	<LOD	<LOD	<LOD
aflatoxin B2	<LOD	<LOD	<LOD	<LOD	<LOD	<LOD	<LOD	<LOD	<LOD
aflatoxin G1	<LOD	<LOD	<LOD	<LOD	<LOD	<LOD	<LOD	<LOD	<LOD
aflatoxin G2	<LOD	<LOD	<LOD	<LOD	<LOD	<LOD	<LOD	<LOD	<LOD
aflatoxin M1	<LOD	<LOD	<LOD	<LOD	<LOD	<LOD	<LOD	<LOD	<LOD
deepoxydeoxynivalenol	<LOD	<LOD	<LOD	<LOD	<LOD	<LOD	<LOD	<LOD	<LOD
deoxynivalenol	<LOD	<LOD	<LOD	<LOD	<LOD	<LOD	<LOD	<LOD	<LOD
deoxynivalenol 3-glucoside	<LOD	<LOD	<LOD	<LOD	<LOD	<LOD	<LOD	<LOD	<LOD
diacetoxyscirpenol	<LOD	<LOD	<LOD	<LOD	<LOD	<LOD	<LOD	<LOD	<LOD
fumonisin B1	<LOD	<LOD	<LOD	<LOD	<LOD	<LOD	<LOD	<LOD	<LOD
fumonisin B2	<LOD	<LOD	<LOD	<LOD	<LOD	<LOD	<LOD	<LOD	<LOD
fumonisin B3	<LOD	<LOD	<LOD	<LOD	<LOD	<LOD	<LOD	<LOD	<LOD
fusarenon X	<LOD	<LOD	<LOD	<LOD	<LOD	<LOD	<LOD	<LOD	<LOD
gliotoxin	<LOD	<LOD	<LOD	<LOD	<LOD	<LOD	<LOD	<LOD	<LOD
griseofulvin	<LOD	<LOD	<LOD	<LOD	1210	<LOD	8220	10900	13600
HT-2 toxin	<LOD	<LOD	<LOD	<LOD	<LOD	<LOD	<LOD	<LOD	<LOD
mevinolin	<LOD	<LOD	<LOD	<LOD	<LOD	<LOD	<LOD	<LOD	<LOD
moniliformin	<LOD	<LOD	<LOD	<LOD	<LOD	<LOD	<LOD	8500	<LOD
monoacetoxyscirpenol	<LOD	<LOD	<LOD	<LOD	<LOD	<LOD	<LOD	<LOD	<LOD
mycophenolic acid	<LOD	<LOD	108.9	237.5	<LOD	1470	<LOD	<LOD	<LOD
neosolaniol	<LOD	<LOD	<LOD	<LOD	<LOD	<LOD	<LOD	<LOD	<LOD
nivalenol	<LOD	<LOD	<LOD	<LOD	<LOD	<LOD	<LOD	<LOD	<LOD
ochratoxin A	<LOD	<LOD	<LOD	<LOD	<LOD	<LOD	<LOD	<LOD	<LOD
ochratoxin B	<LOD	<LOD	<LOD	<LOD	<LOD	<LOD	<LOD	<LOD	<LOD
patulin	<LOD	<LOD	<LOD	<LOD	<LOD	<LOD	366000	380000	30300
roquefortine C	1580	2350	19.4	29690	<LOD	<LOD	<LOD	<LOD	<LOD
sterigmatocystin	<LOD	<LOD	6.658	<LOD	<LOD	<LOD	<LOD	<LOD	<LOD
T-2 toxin	<LOD	<LOD	<LOD	<LOD	<LOD	<LOD	<LOD	<LOD	<LOD
T-2 tetraol	<LOD	<LOD	<LOD	<LOD	<LOD	<LOD	<LOD	<LOD	<LOD
T-2 triol	<LOD	<LOD	<LOD	<LOD	<LOD	<LOD	<LOD	<LOD	<LOD
zearalanone	<LOD	<LOD	<LOD	<LOD	<LOD	<LOD	<LOD	<LOD	<LOD
zearalenone	<LOD	<LOD	<LOD	<LOD	<LOD	<LOD	<LOD	<LOD	<LOD
α-zearalanol	<LOD	<LOD	<LOD	<LOD	<LOD	<LOD	<LOD	<LOD	<LOD
α-zearalenol	<LOD	<LOD	<LOD	<LOD	<LOD	<LOD	<LOD	<LOD	<LOD
β-zearalanol	<LOD	<LOD	<LOD	<LOD	<LOD	<LOD	<LOD	<LOD	<LOD
β-zearalenol	<LOD	<LOD	<LOD	<LOD	<LOD	<LOD	<LOD	<LOD	<LOD

<LOD, below limit of detection; <LOQ, below limit of quantification; values are provided in Table 2.

**Table 7:** Mycotoxins produced by *Aspergillus* sp.

Metabolites	Concentration (ng ^−1^)										
	*A.giganteus* 19cz	*A.sydowii* 5bl	*A. puulaeansis* 4bl	*A. niger* 7f	*A.niger* 15c	*A. niger* 25a	*A. niger* 25cz	*A. westerdijikiae* 11al	*A. westerdijikiae* 1dz	*A. ostianus* 17cj	*A. ostianus* 17cz
15-acetyldeoxynivalenol	<LOD	<LOD	<LOD	<LOD	<LOD	<LOD	<LOD	<LOD	<LOD	<LOD	<LOD
3-acetyldeoxynivalenol	<LOD	<LOD	<LOD	<LOD	<LOD	<LOD	<LOD	<LOD	<LOD	<LOD	<LOD
aflatoxin B1	<LOD	<LOD	<LOD	<LOD	<LOD	<LOD	<LOD	<LOD	<LOD	<LOD	<LOD
aflatoxin B2	<LOD	<LOD	<LOD	<LOD	<LOD	<LOD	<LOD	<LOD	<LOD	<LOD	<LOD
aflatoxin G1	<LOD	<LOD	<LOD	<LOD	<LOD	<LOD	<LOD	<LOD	<LOD	<LOD	<LOD
aflatoxin G2	<LOD	<LOD	<LOD	<LOD	<LOD	<LOD	<LOD	<LOD	<LOD	<LOD	<LOD
aflatoxin M1	<LOD	<LOD	<LOD	<LOD	<LOD	<LOD	<LOD	<LOD	<LOD	<LOD	<LOD
deepoxydeoxynivalenol	<LOD	<LOD	<LOD	<LOD	<LOD	<LOD	<LOD	<LOD	<LOD	<LOD	<LOD
deoxynivalenol	<LOD	<LOD	<LOD	<LOD	<LOD	<LOD	<LOD	<LOD	<LOD	<LOD	<LOD
deoxynivalenol 3-glucoside	<LOD	<LOD	<LOD	<LOD	<LOD	<LOD	<LOD	<LOD	<LOD	<LOD	<LOD
diacetoxyscirpenol	<LOD	<LOD	<LOD	<LOD	<LOD	<LOD	<LOD	<LOD	<LOD	<LOD	<LOD
fumonisin B1	<LOD	<LOD	<LOD	<LOD	<LOD	<LOD	<LOD	<LOD	<LOD	<LOD	<LOD
fumonisin B2	<LOD	<LOD	<LOD	127.9	195.3	30.9	<LOD	<LOD	<LOD	<LOD	<LOD
fumonisin B3	<LOD	<LOD	<LOD	<LOD	<LOD	<LOD	<LOD	<LOD	<LOD	<LOD	<LOD
fusarenon X	<LOD	<LOD	<LOD	<LOD	<LOD	<LOD	<LOD	<LOD	<LOD	<LOD	<LOD
gliotoxin	<LOD	<LOD	<LOD	<LOD	<LOD	<LOD	<LOD	<LOD	<LOD	<LOD	<LOD
griseofulvin	<LOD	<LOD	<LOD	<LOD	<LOQ	<LOD	<LOD	<LOD	<LOD	6.93	10300
HT-2 toxin	<LOD	<LOD	<LOD	<LOD	<LOD	<LOD	<LOD	<LOD	<LOD	<LOD	<LOD
mevinolin	<LOD	<LOD	<LOD	<LOD	<LOD	<LOD	<LOD	<LOD	<LOD	<LOD	<LOD
moniliformin	<LOD	<LOD	<LOD	<LOD	<LOD	<LOD	<LOD	<LOD	<LOD	<LOD	52900
monoacetoxyscirpenol	<LOD	<LOD	<LOD	<LOD	<LOD	<LOD	<LOD	<LOD	<LOD	<LOD	<LOD
mycophenolic acid	<LOD	<LOD	<LOD	158.4	40.9	<LOD	<LOD	<LOD	<LOD	21.8	<LOD
neosolaniol	<LOD	<LOD	<LOD	<LOD	<LOD	<LOD	<LOD	<LOD	<LOD	<LOD	<LOD
nivalenol	<LOD	<LOD	<LOD	<LOD	<LOD	<LOD	<LOD	<LOD	<LOD	<LOD	<LOD
ochratoxin A	<LOD	<LOD	<LOD	<LOD	<LOD	<LOD	23.6	<LOD	<LOD	<LOD	<LOD
ochratoxin B	<LOD	<LOD	<LOD	<LOD	<LOD	<LOD	20.3	<LOD	<LOD	<LOD	<LOD
patulin	84600	<LOD	<LOD	<LOD	<LOD	<LOD	<LOD	<LOD	59.0	<LOD	491000
roquefortine C	<LOD	9.6	<LOQ	21.9	38.4	<LOQ	<LOD	383000	<LOD	<LOQ	<LOD
sterigmatocystin	<LOD	24850	29050	<LOD	<LOD	<LOD	<LOD	<LOD	<LOD	<LOD	<LOD
T-2 toxin	<LOD	<LOD	<LOD	<LOD	<LOD	<LOD	<LOD	<LOD	<LOD	<LOD	<LOD
T-2 tetraol	<LOD	<LOD	<LOD	<LOD	<LOD	<LOD	<LOD	<LOD	<LOD	<LOD	<LOD
T-2 triol	<LOD	<LOD	<LOD	<LOD	<LOD	<LOD	<LOD	<LOD	<LOD	<LOD	<LOD
zearalanone	<LOD	<LOD	<LOD	<LOD	<LOD	<LOD	<LOD	<LOD	<LOD	<LOD	<LOD
zearalenone	<LOD	<LOD	<LOD	<LOD	<LOD	<LOD	<LOD	<LOD	<LOD	<LOD	<LOD
α-zearalanol	<LOD	<LOD	<LOD	<LOD	<LOD	<LOD	<LOD	<LOD	<LOD	<LOD	<LOD
α-zearalenol	<LOD	<LOD	<LOD	<LOD	<LOD	<LOD	<LOD	<LOD	<LOD	<LOD	<LOD
β-zearalanol	<LOD	<LOD	<LOD	<LOD	<LOD	<LOD	<LOD	<LOD	<LOD	<LOD	<LOD
β-zearalenol	<LOD	<LOD	<LOD	<LOD	<LOD	<LOD	<LOD	<LOD	<LOD	<LOD	<LOD

<LOD, below limit of detection; <LOQ, below limit of quantification; values are provided in Table 2.

**Table 8:** Mycotoxins produced by *Aspergillus* sp.

Metabolites	Concentration (ng g^−1^)											
	*A. elegans* 14al	*A. elegans* 14cl	*A. elegans* 24bz	*A. fumigatus* 5bw	*A. fumigatus* 4gw	*A. fumigatus* 3fw	*A. fumigatus* 9bl	*A. fumigatus* 10al	*A. fumigatus* 6cw	*A. fumigatus* 9dl	*A. fumigatus* 10bl	*A. fumigatus* 6dl
15-acetyldeoxynivalenol	<LOD	<LOD	<LOD	<LOD	<LOD	<LOD	<LOD	<LOD	<LOD	<LOD	<LOD	<LOD
3-acetyldeoxynivalenol	<LOD	<LOD	<LOD	<LOD	<LOD	<LOD	<LOD	<LOD	<LOD	<LOD	<LOD	<LOD
aflatoxin B1	<LOD	<LOD	<LOD	<LOD	<LOD	<LOD	<LOD	<LOD	<LOD	<LOD	<LOD	<LOD
aflatoxin B2	<LOD	<LOD	<LOD	<LOD	<LOD	<LOD	<LOD	<LOD	<LOD	<LOD	<LOD	<LOD
aflatoxin G1	<LOD	<LOD	<LOD	<LOD	<LOD	<LOD	<LOD	<LOD	<LOD	<LOD	<LOD	<LOD
aflatoxin G2	<LOD	<LOD	<LOD	<LOD	<LOD	<LOD	<LOD	<LOD	<LOD	<LOD	<LOD	<LOD
aflatoxin M1	<LOD	<LOD	<LOD	<LOD	<LOD	<LOD	<LOD	<LOD	<LOD	<LOD	<LOD	<LOD
deepoxydeoxynivalenol	<LOD	<LOD	<LOD	<LOD	<LOD	<LOD	<LOD	<LOD	<LOD	<LOD	<LOD	<LOD
deoxynivalenol	<LOD	<LOD	<LOD	<LOD	<LOD	<LOD	<LOD	<LOD	<LOD	<LOD	<LOD	<LOD
deoxynivalenol 3-glucoside	<LOD	<LOD	<LOD	<LOD	<LOD	<LOD	<LOD	<LOD	<LOD	<LOD	<LOD	<LOD
diacetoxyscirpenol	<LOD	<LOD	<LOD	<LOD	<LOD	<LOD	<LOD	<LOD	<LOD	<LOD	<LOD	<LOD
fumonisin B1	<LOD	<LOD	<LOD	<LOD	<LOD	<LOD	<LOD	<LOD	<LOD	<LOD	<LOD	<LOD
fumonisin B2	<LOD	<LOD	<LOD	<LOD	<LOD	<LOD	<LOD	<LOD	<LOD	<LOD	<LOD	<LOD
fumonisin B3	<LOD	<LOD	<LOD	<LOD	<LOD	<LOD	<LOD	<LOD	<LOD	<LOD	<LOD	<LOD
fusarenon X	<LOD	<LOD	<LOD	<LOD	<LOD	<LOD	<LOD	<LOD	<LOD	<LOD	<LOD	<LOD
gliotoxin	<LOD	<LOD	<LOD	3490	2820	4190	2155	391.5	<LOD	34300	2555	173.5
griseofulvin	<LOD	<LOD	<LOD	<LOD	<LOD	<LOD	<LOD	<LOD	<LOD	<LOD	<LOD	<LOD
HT-2 toxin	<LOD	<LOD	<LOD	<LOD	<LOD	<LOD	<LOD	<LOD	<LOD	<LOD	<LOD	<LOD
mevinolin	<LOD	<LOD	<LOD	<LOD	<LOD	<LOD	<LOD	<LOD	<LOD	<LOD	<LOD	<LOD
moniliformin	<LOD	<LOD	<LOD	<LOD	<LOD	<LOD	<LOD	<LOD	<LOD	<LOD	<LOD	<LOD
monoacetoxyscirpenol	<LOD	<LOD	<LOD	<LOD	<LOD	<LOD	<LOD	<LOD	<LOD	<LOD	<LOD	<LOD
mycophenolic acid	<LOD	<LOD	<LOD	<LOD	<LOD	<LOD	<LOD	<LOD	<LOD	<LOD	<LOD	<LOD
neosolaniol	<LOD	<LOD	<LOD	<LOD	<LOD	<LOD	<LOD	<LOD	<LOD	<LOD	<LOD	<LOD
nivalenol	<LOD	<LOD	<LOD	<LOD	<LOD	<LOD	<LOD	<LOD	<LOD	<LOD	<LOD	<LOD
ochratoxin A	<LOD	530	203	<LOD	<LOD	<LOD	<LOD	<LOD	<LOD	<LOD	<LOD	<LOD
ochratoxin B	<LOD	247.5	79.3	<LOD	<LOD	<LOD	<LOD	<LOD	<LOD	<LOD	<LOD	<LOD
patulin	<LOD	<LOD	<LOD	<LOD	<LOD	<LOD	<LOD	<LOD	<LOD	<LOD	<LOD	<LOD
roquefortine C	65000	25.0	<LOD	<LOD	<LOD	<LOD	<LOD	<LOQ	348000	<LOD	18.6	<LOD
sterigmatocystin	<LOD	<LOD	<LOD	<LOD	<LOD	<LOD	<LOD	<LOD	<LOD	<LOD	<LOD	<LOD
T-2 toxin	<LOD	<LOD	<LOD	<LOD	<LOD	<LOD	<LOD	<LOD	<LOD	<LOD	<LOD	<LOD
T-2 tetraol	<LOD	<LOD	<LOD	<LOD	<LOD	<LOD	<LOD	<LOD	<LOD	<LOD	<LOD	<LOD
T-2 triol	<LOD	<LOD	<LOD	<LOD	<LOD	<LOD	<LOD	<LOD	<LOD	<LOD	<LOD	<LOD
zearalanone	<LOD	<LOD	<LOD	<LOD	<LOD	<LOD	<LOD	<LOD	<LOD	<LOD	<LOD	<LOD
zearalenone	<LOD	<LOD	<LOD	<LOD	<LOD	<LOD	<LOD	<LOD	<LOD	<LOD	<LOD	<LOD
α-zearalanol	<LOD	<LOD	<LOD	<LOD	<LOD	<LOD	<LOD	<LOD	<LOD	<LOD	<LOD	<LOD
α-zearalenol	<LOD	<LOD	<LOD	<LOD	<LOD	<LOD	<LOD	<LOD	<LOD	<LOD	<LOD	<LOD
β-zearalanol	<LOD	<LOD	<LOD	<LOD	<LOD	<LOD	<LOD	<LOD	<LOD	<LOD	<LOD	<LOD
β-zearalenol	<LOD	<LOD	<LOD	<LOD	<LOD	<LOD	<LOD	<LOD	<LOD	<LOD	<LOD	<LOD

<LOD, below limit of detection; <LOQ, below limit of quantification; values are provided in Table 2.

## Data Availability

The original contributions presented in the study are included in the article; further inquiries can be directed to the corresponding author/s.

## Supplementary Materials

The following supporting information can be downloaded at: https://www.mdpi.com/article/10.3390/pathogens13040294/s1, Table S1. BLAST analysis of the ITS rDNA of fungi found in a zoological garden. All E values were set to zero.
