# Supplementary material for: Analysis of Mycotoxins and Cytotoxicity of Airborne Molds Isolated from the Zoological Garden—Screening Research
Source: Pathogens. 2024 Mar 30;13(4):294. doi: 10.3390/pathogens13040294 (PMC11053870; doi:10.3390/pathogens13040294)
Supplement: Supplementary file 1 [file pathogens-13-00294-s001.zip › pathogens-2920382-supplementary.pdf]

Supplemental Table S1. BLAST analysis of the ITS rDNA of fungi found in a zoological garden. All E values were set to zero.

Fungal Species	Isolate	GenBank Accession No	Identity with Sequence from GenBank	
			Identity	Accession No
Aspergillus niger	7fj	OR807891	100%	MT620753.1
Aspergillus niger	15cj	OR807968.1	100%	MT620753.1
Aspergillus niger	25aj	PP494172	100%	MT620753.1
Aspergillus ochraceus	3bz	OR801670.1	100%	OL691169.1
Aspergillus westerdijkiae	1dz	OR816133.1	99.55%	MT635281.1
Aspergillus ostianus	17cj	OR801711.1	100%	ON207625.1
Aspergillus elegans	14al	PP494195	100%	MN886600.1
Aspergillus elegans	24bz	PP494173	100%	OR594271.1
Aspergillus elegans	22cz	OR802142	100%	MN886600.1
Aspergillus flavus	2gw	OR806964.1	100%	OR397999.1
Aspergillus flavus	18aw	OR806964.1	100%	MT528892.1
Aspergillus giganteus	19cz	OR808008	100%	MT529982.1
Aspergillus sydowii	4bl	OR807981.1	100%	OR807981.1
Aspergillus niger	25cz	PP494174	99.82%	MT628904.1
Aspergillus fumigatus	3fw	OR8069661	100%	MN559667.1
Penicillium commune	22az	PP494181	100%	KY606533.1
Penicillium commune	17fz	PP494180	100%	MT378399.1
Penicillium solitum	15aj	OR810004.1	99.63%	OM959583.1
Penicillium raistrickii	9bw	OR810006.1	100%	MK450710.1
Penicillium glandicola	6aw	OR810010.1	100%	MH860946.1
Penicillium griseofulvum	17az	OR810001.1	100%	MT378399.1
Penicillium griseofulvum	18cz	PP494182	100%	MT378399.1
Penicillium griseofulvum	19dz	OR810000.1	100%	MT378399.1
Penicillium chrysogenum	20 Aez	PP494176	100%	MT229079.1
Penicillium chrysogenum	15aw	OR810002.1	100%	JQ082504.1
Penicillium glabrum	1bw	PP494188	100%	MN856208.1
Penicillium citreosulfuratusm	1az	OR810011.1	100%	MN592912.1
Penicillium citrinum	15az	OR810014.1	100%	MT597829.1
Penicillium citrinum	19bz	PP494183	100%	JN859855.1
Penicillium citrinum	15dl	PP494191	100%	JN859855.1
Penicillium citrinum	15bl	OR810013.1	99%	MK451671.1
Penicillium steckii	18cw	OR810015.1	100%	MK805469.1
Penicillium steckii	20cz	OR810016.1	100%	MT582790.1
Penicillium steckii	18bz	PP494177	100%	MK805469.1
Penicillium sumatraense	15bz	PP494184	100%	MT582791.1
Penicillium sumatraense	16dw	PP494190	99.15%	MK910053.1
Penicillium brevicompactum	2bj	PP494189	100%	MT558924.1
Penicillium bialowieziense	9bj	OR810007.1	100%	MT582764.
Penicillium olsonii	13bz	PP494186	100%	MT133794.1
Penicillium copticola	17bz	PP494185	100%	MH864539.1
